# Comparative inner ear transcriptome analysis between the Rickett’s big-footed bats (*Myotis ricketti*) and the greater short-nosed fruit bats (*Cynopterus sphinx*)

**DOI:** 10.1186/1471-2164-14-916

**Published:** 2013-12-23

**Authors:** Dong Dong, Ming Lei, Yang Liu, Shuyi Zhang

**Affiliations:** 1 Institute for Advanced Interdisciplinary Research in Science and Technology, East China Normal University, Shanghai, China

## Abstract

**Background:**

Bats have aroused great interests of researchers for the sake of their advanced echolocation system. However, this highly specialized trait is not characteristic of Old World fruit bats.

**Results:**

To comprehensively explore the underlying molecular basis between echolocating and non-echolocating bats, we employed a sequence-based approach to compare the inner ear expression difference between the Rickett’s big-footed bat (*Myotis ricketti*, echolocating bat) and the Greater short-nosed fruit bat (*Cynopterus sphinx*, non-echolocating bat). *De novo* sequence assemblies were developed for both species. The results showed that the biological implications of up-regulated genes in *M. ricketti* were significantly over-represented in biological process categories such as ‘cochlea morphogenesis’, ‘inner ear morphogenesis’ and ‘sensory perception of sound’, which are consistent with the inner ear morphological and physiological differentiation between the two bat species. Moreover, the expression of *TMC1* gene confirmed its important function in echolocating bats.

**Conclusion:**

Our work presents the first transcriptome comparison between echolocating and non-echolocating bats, and provides information about the genetic basis of their distinct hearing traits.

## Background

Bats belong to the order Chiroptera, which is one of the largest monophyletic clades in mammals. They constitute ~20% of living mammalian species with about 1,200 bat species worldwide [1-3]. Bats have long been regarded as special animals for the sake of being mysterious flyers of the night, and one of the few mammals that can use echolocation to navigate in the dark [4]. Although many mammalian species are capable of detecting ultrasonic sounds (>20 kHz), only toothed whales and laryngeal echolocating bats have the most advanced auditory systems for detecting ultrasonic sound [5]. Laryngeal echolocating bats can emit broad ultrasound frequency sounds and listen to their echoes, allowing them to orient in the dark and exploit food sources of the night sky. However, not all bats have laryngeal echolocation ability. Traditionally, bats can be divided into two suborders, the ‘Megachiroptera’ (megabats) and the ‘Microchiroptera’ (microbats), identified mainly on the basis of their morphology and echolocation characteristics [2]. Megachiroptera, consisting of a single family, is a smaller group of bats that mainly live in the Old World tropical places. They have good dim light vision and do not have laryngeal echolocation ability, whereas Microchiropteran bats can use sophisticated laryngeal echolocation for orientation [6,7].

With special morphological and physiological adaptations, echolocation allows bats to listen for their high frequency echoes to locate their prey in the dark. The cochleae of these bats are well-developed and show adaptations for detecting their own ultrasonic sounds [5]. The cochlea is a key auditory system of the inner ear and is specialized for the use of high frequency sounds in the echolocating bats. It has been documented that the variation of cochlear size is associated with echolocation strategies [8,9], and the cochlea diameters of laryngeal echolocating bats are higher than those of many non-echolocating megabats [8,10,11]. These phenotypic adaptations are thought to play important roles in the reception and funneling of high frequency echoes. Laryngeal echolocation is a highly technical and physiological adaption, however the molecular basis responsible for this phenotype is poorly characterized for the differences between echolocating and non-echolocating bats.

Divergence of gene expression is an important component of species evolution and essential means to generate biological diversity [12]. Recent next-generation sequencing technologies provided us a large-scale platform to address evolutionary questions involving non-model organisms for which there are still limited genomic resources [13,14]. In this work, we explored the utility of next-generation sequencing technologies for the comparative inner ear transcriptome analyses between the Rickett’s big-footed bat (*Myotis ricketti*) and the Greater short-nosed fruit bat (*Cynopterus sphinx*). The Rickett’s big-footed bats are species of microbats in the family Vespertilionidae. They have advanced laryngeal echolocation ability, which belongs to frequency modulated type; the Greater short-nosed fruit bats are species of megabats and come from the family Pteropodidae without laryngeal echolocation. Based on more recent molecular phylogenetic studies, the divergence time between these two bat species is around 60 million years ago (MYA) [3,15]. The genome sequences of these two bat species are still lacking, and we employed a *de novo* assembly approach to gain insight into the genome-wide expression divergence patterns between the Rickett’s big-footed bat and Greater short-nosed fruit bat. The main purpose of this work is to elucidate the nature of transcriptomes of bat species and determine to what extent the two bat species with different hearing traits differ in gene expression. This study provided an initial step to comprehensively understand the inner ear transcriptome involved in the bat echolocation.

## Results

### *De novo* assembly and functional annotation

Sequencing of the mRNA in the inner ear of Rickett’s big-footed bat (*M. ricketti*, echolocating bat) and the Greater short-nosed fruit bat (*C. sphinx*, non-echolocating bat) based on Illumina Genome Analyzer II generated a total of 6.8 Gbp of sequence from approximately 90 million paired-end 75 bp reads (3.3 GB and 3.5 GB for *M. ricketti* and *C. sphinx*, respectively).

**Figure 1:**
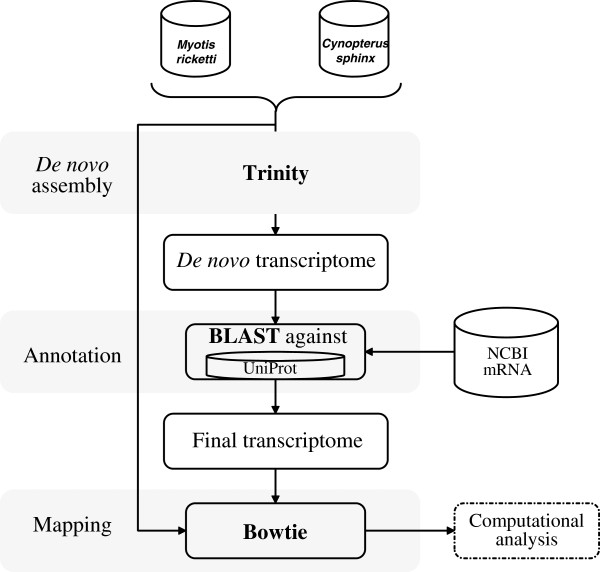
***De novo *assembly workflow.** The figure summarizes computational procedures for assembly, annotation and mapping of the sequencing reads.

**Figure 2:**
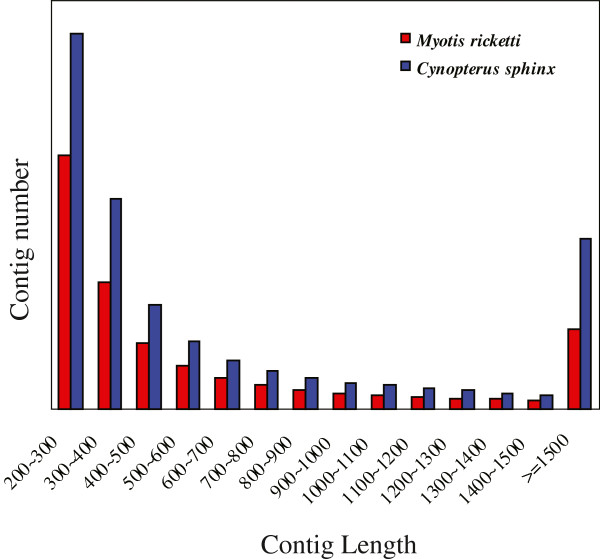
**Length distribution of distinct our assembled contig sequences.** The red and blue bars represent the distribution of contig size for *de novo* assemblies from sequencing reads of *M. ricketti* and *C. sphinx*, respectively.

Because no reference genomes was available for *M. ricketti* and *C. sphinx*, we employed a bioinformatics *de novo* assembly method (Figure 1) in our work. After trimming the adapter sequences and removing sequences with low quality, we used Trinity software [16] to generate *de novo* assemblies of each species, resulting in 104,987 and 171,394 non-redundant contigs in *M. ricketti* and *C. sphinx*, respectively. The detailed assembly results are summarized in Table 1. Next, we performed an analysis of the length distribution of these assembled contigs in two bat species. We excluded all contigs with the length shorter than 200 bp from further analysis because of their short length rendering them useless for most applications. As shown in Figure 2, nearly 50% of the contigs are between 200 to 350 bp, and we even identified 11,717 and 25,048 contigs with their lengths longer than 1,500 bp in *M. ricketti* and *C. sphinx*. To assess the quality of our assembled contigs, we downloaded all cDNA sequences (820 in total) of *M. ricketti* and *C. sphinx* from NCBI Genbank database (non-redundant nt database, downloaded on April 13, 2013) and regarded them as reference sequences. We compared our assembled contigs against these reference sequences using BlastN (E-value < 1e-10), and the best-hit contigs were aligned using Mafft [17]. The fractions of mismatching nucleotides were subsequently calculated. We found that assembly error rates are only ~ 2% relative to the reference transcripts. Our result demonstrated that the assembled sequences of short-read sequences have considerable utility for comparative genomic analysis.

**Table 1:** **Summary of the inner ear transcriptome sequencing assemblies in *M. ricketti *** **and *C. sphinx***

	***M. ricketti***	***C. sphinx***
Raw reads	42862640	48167814
Filtered reads	42404611	47749665
Reads used	31143451	34402281
N50	1135	1493
Max contig length	14199	18746
Mean contig length	697	810
Number of contigs	104987	171394
Number of contigs (> = 1 k bp)	19684	38833

**Figure 3:**
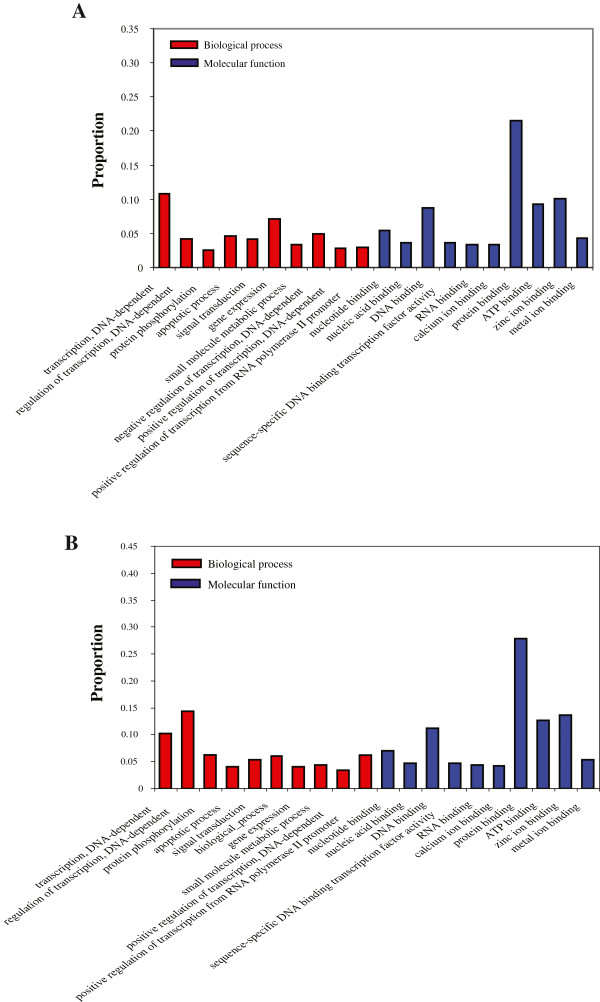
**Distribution of annotation genes at GO sub-ontologies of ‘biological process’ and ‘molecular function’. A***M. ricketti***B***C. sphinx.*

Next, a total of 32,892 (34%, *M. ricketti*) and 44,866 (29%, *C. sphinx*) contigs were aligned against the non-redundant UniProt database using BLAST (E-value < 1E-6). We used Gene Ontology (GO) categories from database to assign their putative biological functions. To improve the GO annotation quality, we only selected the GO annotations that are based upon direct experimental evidence codes. As a result, 12,427 and 13,539 transcripts were annotated with at least one GO functional category in *M. ricketti* and *C. sphinx*, respectively. These two bat species represent similar GO functional annotations. Among the broad GO category ‘biological process’, regulation of transcription, DNA-dependent (GO:0006355) and transcription, DNA dependent (GO:0006351) were most highly represented. In addition, of the transcripts annotated with ‘molecular function’ terms, the most represented categories were ‘protein binding’ (GO:0005515) and ‘zinc ion binding’ (GO:0008270) (Figure 3).

### Transcripts differentially expressed between *M. ricketti* and *C. sphinx*

**Figure 4:**
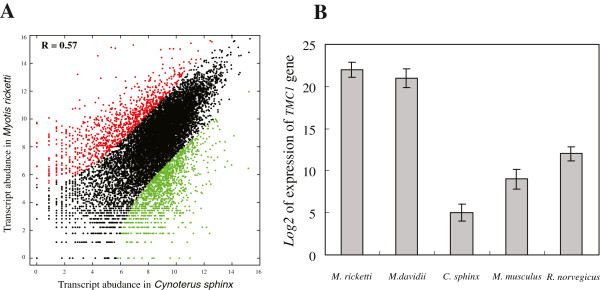
**Gene expression in M. ricketti and C. sphinx. A** Pairwise comparisons of transcript abundances between *M. ricketti* and *C. sphinx*. Each dot represents one orthologous genes between *M. ricketti* and *C. sphinx.* Orthologous genes exhibiting significant up-regulated expression in *M. ricketti* are provided in red, whereas orthologous genes exhibiting significant up-regulated expression in *C. sphinx* are provided in green. Orthologous genes with similar expression in the compared libraries are shown in black. **B** Comparison of expression levels of *TMC1* gene in *M. ricketti*, *M. davidii*, *C. sphinx*, *M. musculus* and *R. norvegicus*.

To investigate the expression patterns of the inner ear in bats, we separately evaluated the number of reads assembled from each library for each transcript and compared gene expression difference between *M. ricketti* and *C. sphinx*. Because of the differences in library size (i.e. sequencing depth), inter-sample normalization was performed at first. Transcript abundances were quantified using RSEM software [18] in which a generative statistical model is used to solve the problem of read mapping uncertainty. In this work, EBSeq method was used [18,19] to calculate the scaling factor and gauge gene expression differences between *M. ricketti* and *C. sphinx*. As expected, we found significant correlation in expression between these two samples (r = 0.57, Figure 4A). A total of 2,272 genes were identified to be significantly differentially expressed between *M. ricketti* and *C. sphinx* among 11,717 orthologous genes, and there were 987 genes that were significantly highly expressed in the inner ear of *M. ricketti*. The number of differentially expressed genes being up-regulated in the non-echolocating bat (1,285 genes) is higher than the number of genes being up-regulated in the echolocating bat (987 genes), indicating that echolocation is not simply a problem of numbers of highly expressed genes, but might confine to only a few gene categories.

To gain insights into biological implications of differentially expressed genes, we performed a GO enrichment analysis for the gene set. GO terms are organized into three sub-ontologies: ‘biological process’, ‘molecular function’ and ‘cellular component’ [20]. The three categories characterize different aspects of gene’s function and only the categories of ‘biological process’ and ‘molecular function’ are examined in our work. In the up-regulated genes in the *C. sphinx*, there are six over-represented GO categories, some of them related to regulation of cell cycle and development (Table 2). In the up-regulated genes in *M. ricketti*, there are seven significantly enriched GO categories in terms of ‘biological process’ and three GO categories in terms of ‘molecular function’ (Table 2). Interestingly, we found three GO categories related to auditory organs development or auditory process (cochlea morphogenesis GO:0090103, inner ear morphogenesis GO:0042472, sensory perception of sound GO:0007605). We defined the genes in these three GO categories as hearing-related genes.

**Table 2:** **Significantly enriched GO terms of differentially expressed genes between *M. ricketti *** **and *C. sphinx***

		**GO ID**	**GO term**	**No. of genes**	***F.D.R***
Up-regulated genes in the *M. ricketti*	BP	GO:0030049	Muscle filament sliding	15	1.66E-10
		GO:0006936	Muscle contraction	17	3.92E-05
		GO:0051216	Cartilage development	13	3.24E-04
		GO:0006941	Striated muscle contraction	7	1.22E-03
		GO:0090103	Cochlea morphogenesis	7	4.96E-03
		GO:0007605	Sensory perception of sound	18	8.33E-05
		GO:0042472	Inner ear morphogenesis	11	7.81E-04
	MF	GO:0008307	Structural constituent of muscle	16	6.70E-04
		GO:0031432	Titin binding	6	4.96E-05
		GO:0001158	Enhancer sequence-specific DNA binding	5	5.37E-04
Up-regulated genes in the *C. sphinx*	BP	GO:0000087	M phase of mitotic cell cycle	21	6.67E-04
		GO:0006271	DNA strand elongation involved in DNA replication	12	1.74E-03
		GO:0000278	Mitotic cell cycle	48	1.69E-03
		GO:0001556	Oocyte maturation	6	2.88E-03
	MF	GO:0005543	Phospholipid binding	51	1.56E-03
		GO:0008270	Zinc ion binding	144	6.23E-03

Note: ‘BP’ and ‘MF’ represent GO categories of biological process and molecular function, respectively.

Based on the alignments of 11,717 orthologous genes, *d*_*N*_*/d*_*S*_ values were estimated between two bat species. *d*_*N*_*/d*_*S*_ > 1 indicates that strong positive selection has acted to change the sequence, while lower *d*_*N*_*/d*_*S*_ value means a more conservative trademark. The average *d*_*N*_*/d*_*S*_ rate for all orthologous genes is 0.18 ± 0.013 (mean ± SD). When considering hearing related genes, the average *d*_*N*_*/d*_*S*_ rate genes is 0.12 ± 0.041(mean ± SD), suggesting that hearing related genes are under strong functional constraint. We next examined the relationships between expression divergence and sequence evolution, and found the expression divergence was largely independent from the sequence evolution (*r* = 0.00017, *P* = 0.9). However, the expression divergences of hearing related genes are negatively correlated with sequence evolution (*r* = -0.16, *P* < 1e-23).

### Quantitative RT-PCR analysis of *TMC1* gene in five mammalian species

Our study comprehensively proved that there are dramatic expression divergence of the inner ear between the echolocating bats and non-echolocating bats. Echolocating bats underwent a unique suite of cochlear morphological modifications that enabled them to receive their high-frequency ultrasonic echoes. Our results are in line with previous morphological work that the cochlear apparatus in the inner ear is proportionately larger in the echolocating bats than in other animals [21]. The genes related to cochlear morphogenesis are preferentially expressed in the inner ear, which makes it possible for the echolocating bats to achieve great hearing sensitivity and perception. Notably, we found that 18 hearing related genes (‘sensory perception of sound’ GO:0007605, Table 3) have significantly higher expression level in the echolocating bat. Among these hearing related genes, *TMC1* gene showed the most significant difference between *M. ricketti* and *C. sphinx*, with a ~57 fold higher expression in *M. ricketti. TMC1* encodes transmembrane protein of the inner ear acting as an intracellular regulatory signal during the hair cell maturation [22]. Recent works have documented that *TMC1* gene underwent positive Darwin selection in the lineage leading to *Myotis lucifugus*[23] and *Myotis davidii*[24], which indicated that *TMC1* gene might be an important bat echolocating related gene. In order to validate the expression level of *TMC1* gene in the inner ear and extend to other species, Real-Time PCR was then adopted to five mammalian species (*M. ricketti*, *M. davidii*, *C. sphinx*, *M. musculus* and *R. norvegicus*). *TMC1* mRNA levels vary in different species, and it showed significantly higher expression level in the *M. ricketti* and *M. davidii* compared with the *C. sphinx*, *M. musculus* and *R. norvegicus* (*P-value* < 1e-6 Figure 4B). *M. ricketti* and *M. davidii* are all laryngeal echolocating bats having the most sensitive hearing at high frequencies, and *TMC1* gene of these two laryngeal echolocating bats have the highest expression level than the non-echolocating bat and two rodents.

**Table 3:** **The significantly up-regulated hearing related genes in *M. ricketti***

**Gene symbol**	**Description**	**Transcript abundance ( *M.ricketti *)**	**Transcript abundance ( *C.sphinx *)**	***FDR***
*TMC1*	Transmembrane channel-like 1	552.29	9.66	6.7E-19
*LOXHD1*	Lipoxygenase homology domains 1	445.47	14.94	2.1E-16
*OTOS*	Otospiralin	33193.28	1244.82	5.5E-12
*OTOG*	Otogelin	6776.79	355.90	6.9E-07
*COL11A2*	Collagen, type XI, alpha 2	761.07	51.08	1.8E-06
*USH1C*	Usher syndrome 1C (autosomal recessive, severe)	1718.78	131.81	7.9E-06
*CEACAM16*	Carcinoembryonic antigen-related cell adhesion molecule 16	2238.29	182.07	8.6E-06
*TBX1*	T-box 1	810.84	75.79	9.1E-06
*CASP3*	Caspase 3, apoptosis-related cysteine peptidase	1275.73	135.93	8.8E-05
*GJB2*	Gap junction protein, beta 2, 26 kDa	12476.91	1249.77	6.3E-05
*GJB6*	Gap junction protein, beta 6, 30 kDa	8504.06	895.52	2.2E-05
*CHRNA9*	Cholinergic receptor, nicotinic, alpha 9 (neuronal)	83.75	2.47	2.9E-05
*POU3F4*	POU class 3 homeobox 4	194.21	18.12	9.6E-04
*NDUFB9*	NADH dehydrogenase (ubiquinone) 1 beta subcomplex, 9, 22 kDa	9918.17	1207.75	5.3E-04
*MYO3A*	Myosin IIIA	118.95	9.89	4.2E-04
*SIX1*	SIX homeobox 1	3038.21	490.19	6.7E-03
*SOX2*	SRY (sex determining region Y)-box 2	485.53	81.56	3.2E-03
*HEXA*	Hexosaminidase A (alpha polypeptide)	1670.22	298.23	6.2E-03

## Discussion

We have performed the novel inner ear transcriptome of two bat species (*M. ricketti* and *C. sphinx*) using Illumina sequencing technology. As was first proposed for over several decades ago, alterations (or innovations) in gene expressions were regarded as essential means to generate biological diversity [12,25]. The characterization of transcriptome is essential to distinguish the functional implications of different species and to obtain a better understanding of their biological complexity. So, the analysis of differentially expressed genes can elucidate the molecular mechanisms underlying the morphological diversity and provide a better understanding of the relationship between gene expression patterns and the resultant morphologies. To our knowledge, this work represents the first effort to comparatively analyze the inner ear transcriptome of the bat species resulting in a reference transcriptome of more than 16,000 annotated genes from the *M. ricketti* and *C. sphinx* which represent the echolocating bat and non-echolocating bat, respectively.

Our transcriptome work based on next-generation sequencing technology attempted to reveal the underlying molecular mechanisms of the auditory system between the echolocating bat and non-echolocating bat at transcription level. Compared with other next-generation sequencing technology, such as Roche 454 technology, the Illumina solexa platform offers a higher sequencing depth with considerably less cost, which ensures more complete coverage of the transcriptome. In this work, 104,987 and 171,394 contigs were assembled for *M. ricketti* and *C. sphinx,* respectively. We evaluated the quality and quantity of short-read assemblies and proved that short-reads transcriptome assemblies are large in quantity and high in quality for further analysis, which can provide considerable utilities for non-model organisms.

Bats are amongst the few mammal species that use sophisticated echolocation. The generated high-frequency calls bounces off surrounding objects and the returning echoes allows them for the detection, localization and classification of these objects with extraordinary acuity. This astonishing sensory ability enables bats to navigate in the dark. Although bats are not the only mammals to have evolved ultrasonic echolocation, they are certainly the most developed species of the laryngeal echolocation. Fossil evidence suggests that bats might echolocate even at an early stage of their evolution [26]. However, none of the Old World fruit bats have laryngeal echolocation ability, and they use their sense of vision to locate food [6]. As we know, the sound waves enter the ear and travel until they reach the middle ear. The auditory ossicles will vibrate as a response to the sound waves, and the stirrup bone transmits vibrations into the inner ear. Then, the pressure waves flatten the hair of the inner ear and made them perceived as a sound. The inner ear consists of a cochlea which is a spiral-shaped cavity that functions as sound reception and processing apparatus for hearing. Cochlea functions as sound reception and processing apparatus in the inner ear, and its size is known to be correlated with echolocation behavior, and the cochlea structure significantly contributed to the diversification of bat species [26]. A recent work reconstructed three-dimensional inner ear volumes of both echolocating and non-echolocating bats, and found that hearing in bats correlated with both measures of cochlea morphology [9]. In this work, we evaluated and compared the expression difference between echolocating and non-echolocating bats, and found that hearing-related genes show significantly differential expression. Organs in the body always exhibit specialized forms that are essential for their functions. Although bat echolocation calls show a great diversity in duration and shape, all echolocating bats have specialized inner ears which allow them to hear sounds in the ultrasonic range. The expressions of inner ear/cochlea morphogenesis genes are responsible for the senses of hearing and balance, which might associate with high-frequency hearing ability. Based on morphological and anatomical analyses, the cochlea is enlarged relative to other skull structures in the echolocating bats, which makes them able to detect, and discriminate between high-frequency calls, whereas non-echolocating bats tend to have smaller cochleae than bats that use laryngeal echolocation [27]. As we know, the generation of animal inner ear requires coordination between morphogenesis and cell fate specification [28]. The inner ear/cochlea morphogenesis genes are highly expressed in echolocating bats, which are consistent with these findings that the cochlea of echolocating bats is enlarged relative to other skull structures.

Most previous works focused on morphological comparison between echolocating bats and non-echolocating bats. It has been documented that gene expression differences might be important contributors to echolocation-specific features [29,30]. We compared the gene expression divergence between *M. ricketti* and *C. sphinx*, and aimed to put these expression differences in context by comparing them to the high-frequency hearing traits. Of the two bats we examined, the inner ear of *M. ricketti* was found to have higher expression level of hearing related genes and inner ear morphogenesis genes, such as *TMC1* gene, which is probably an adaptation to their developed auditory tuning to their high-frequency calls. *TMC1* gene is involved in hair cell structure and function, and show evidences of positive selection exerted on the some echolocating bat species [23,24]. Thus, the study of the development of bat inner ear is important to understand the molecular mechanisms underlying the generation of ear with high-frequency hearing ability. We previously demonstrated the importance of *Prestin* gene in high frequency hearing mammals [31-33]. However, *Prestin* was shown a low expression value in both species, and no significant expression difference was found between *M. ricketti* and *C. sphinx* in this work (13.4 *vs.* 10.7, *P-value* = 0.21). Although the sequence evolution of *Prestin* contributes to the evolution of bat hearing, the result shows that echolocation of bats isn’t associated with the transcription level of *Prestin* gene.

It has been realized that stabilizing selection is likely to be the dominant signature of expression evolution [34]. Although the expression evolution between two species involves different habitat use or ecological niches, the mode of expression divergence is largely explained by neutral evolution and not of direct adaptive significance. Moreover, the neutral view of gene expression divergence asserts that the functionally irrelevant component of gene expression evolves neutrally. Based on this scenario, the vast majority of gene expression divergences between two species should reflect neutral (as opposed to adaptive) variations. Based on this principle, most of expression changes between two species are likely to be of no direct significance. However, high-throughput expression analyses of the inner ear have shown that abnormal events of gene expression of deafness genes in the inner ear is associated with a varying degree of hearing loss [35]. Furthermore, the finding of the enlarged cochlea relative to other skull structures in the echolocating bat can be well explained by the elevated expression of cochlea/inner ear morphogenesis genes. Although we still cannot accurately describe a more precise expression evolution trajectory history at the current form, our work provides a starting point for experimental follow-up.

## Conclusion

The study of inner ear gene expression divergences between *M. ricketti* and *C. sphinx* using next generation sequencing technology revealed the extent of inner ear transcriptome evolution between the echolocating and non-echolocating bat*,* and identified a number of novel candidate genes associated with the echolocation ability. Revealing the difference of auditory systems between echolocating bats and non-echolocating bats not only provided better insight into understanding the causes of high-frequency sound hearing ability of echolocating bats, but has also opened an opportunity into exploring how the echolocation evolved in bats.

## Methods

### Sampling, RNA extraction and sequencing

All procedures were in accordance with the guidelines of Regulations for the Administration of Laboratory Animals (Decree No. 2 of the State Science and Technology Commission of the People’s Republic of China on November 14, 1988) approved by the Animal Ethics Committee of East China Normal University (ID no: 20090219). We captured the Rickett’s big-footed bats from a cave (39°42′N, 115°43′E) in Beijing in Oct. 2009, and the greater short-nosed fruit bats were captured during the period of Oct. 2009 from Yuexiu park (23°08′N, 113°20′E) located at Guangdong province, China. The inner ear of the Rickett’s big-footed bats and the Greater short-nosed fruit bats were collected. All tissues were flash frozen in liquid nitrogen and placed in a -80°C freezer until processed for total RNA isolation. Total RNA was isolated using TRIzol (Life Technologies Corp., Carlsbad, CA, USA) according to the manufacturer’s protocols and cleaned up using the RNeasy mini kit (Qiagen, Valencia, CA, USA). RNA samples were quantified by the 2100 Bioanalyzer (Agilent Technologies). We purified mRNA using RNA-Seq sample preparation Kit (Illumina, San Diego, CA). Four paired-end cDNA libraries of each tissue were generated using mRNA-Seq assay for transcriptome sequencing on Illumina Genome Analyzer II platform. Short sequence reads of 75 bp were generated. All these data have been deposited into the NCBI Sequence Read Archive database (SRA run accession numbers: *M. ricketti*: # SRR837386 and *C. sphinx*: #SRR837385).

### *De novo* assembly

At first, we removed the low quality reads prior to analyzing the data. Two criteria were used in this filtering step: removing reads with adaptors; removing reads with unknown ‘N’ bases. All subsequent analyses were based on these filtered reads. Next, *de novo* sequence assembly was carried out using Trinity software [16] designed for short read sequences assembly with default parameters. Only contigs with length greater than 200 bp were used for further analysis. To lower the redundancy in the dataset, low-coverage artifacts or redundancies were removed by using CD-HIT [36] with an identity threshold of 95%. The detailed work flow is described in Figure 1. To assess the quality of our assemblies, we downloaded all cDNA sequences (820 in total) of *M. ricketti* and *C. sphinx* from NCBI Genbank database, we searched the online Genbank database (non-redundant *nt* database) using species names and downloaded them in bulk (downloaded on April 13, 2013).

### Functional annotation and identification of orthologous

Assembled contigs were annotated by using the best hits of BLASTX search against the non-redundant UniProt database with an E-value cutoff of 1E-6 for the annotation of these protein coding contigs that were conserved with other species. Next, we performed a pairwise multiple alignment was performed and the contigs with their the percentage of identity lower than 50% were discarded. The results of the best blast hits were extracted, and the open reading frames were subsequently determined. Next, a reciprocal best BLASTP search was conducted. The Gene Ontology (GO) categories for the non-redundant UniProt proteins (Release 2013 05) was used to assign the GO terms to these transcripts. In our study, only experiment based GO annotations were applied, which can provide a better quality data.

After the differentially expressed genes were classified into different GO categories, an in-house fisher’s exact test program was used to map differentially expressed genes to GO terms. The calculated *P values* were corrected through Bonferroni correction, and the corrected *P values* (false discovery rate, *F.D.R*) were taken as the thresholds of significance.

### Measurement of gene expression

To estimate gene expression level, we measured the reads number derived from each contig or isoform using RSEM software package [18]. Next, EBSeq method nested in RSEM package was performed to detect the differentially expressed genes between these two bat species. EBSeq is a Bayesian hierarchical model for the inference on the differential gene expression based on RNA-Seq data [19]. We adjusted the significant level by a correction for false discovery rate (FDR) at < 0.01 with Benjamini-Hochberg correction method [37].

### Estimation of substitution rates

Pair-wise and multiple alignments were generated for two bat species using Mafft software [17] based on protein sequences and back –translated to DNA sequences. We estimated the overall substitution rates (non-synonymous substitution to synonymous substitution, *d*_*N*_*/d*_*S*_) using a maximum likelihood method implemented in the CODEML program nested in PAML package version 4.1 [38]. To minimize statistical artefacts from short sequences and saturation effects in *d*_*S*_ value, we excluded all alignments that were shorter than 100 bp or that *d*_*S*_ value larger than 2 from the analysis.

### Quantitative RT-PCR

To validate the expression patterns observed from our RNA-seq analyses, three replicates were analyzed per species using quantitative polymerase chain reaction (qPCR) using the SYBR Prime-Script RT-PCR Kit (TaKaRa) on Applied Biosystems 7300 Real-Time PCR System (Applied Biosystems). We sampled the inner ears from three bat species (*M. ricketti*, *M. davidii*, *C. sphinx*) and two rodents (*M. musculus* and *R. norvegicus*). Total RNA was extracted using TRIzol Reagent (Invitrogen) and treated with DNase I (Roche). PCR products were purified and sequenced in both directions with an ABI 3730 DNA sequencer (Applied Biosystems). We designed gene specific primers of *TMC1* gene for qPCR. Student’s t-test was performed to examine the difference of gene expression between two different samples. Sequences of forward and reverse primers for normal PCR and qPCR are shown in Additional file 1: Table S1.

## Competing interests

The authors declare that they have no competing interests.

## Authors’ contribution

DD designed the experiments. DD, ML,YL and SZ analyzed the data. DD wrote the paper. All authors read and approved the final manuscript.

## Supplementary Material

Additional file 1: Table S1 Primer sequences for TMC1 gene. Click here for file
