# Supplementary material for: Comparative inner ear transcriptome analysis between the Rickett’s big-footed bats (Myotis ricketti) and the greater short-nosed fruit bats (Cynopterus sphinx)
Source: BMC Genomics. 2013 Dec 23;14:916. doi: 10.1186/1471-2164-14-916 (PMC3879654; doi:10.1186/1471-2164-14-916)
Supplement: Additional file 1: Table S1 — Primer sequences for TMC1 gene. [file 1471-2164-14-916-S1.doc]

**Table S1**

| **Number of**  **Primers** | **Primer sequences** |
| --- | --- |
| 1 | F5’-3’ 5’ GCAATTGAAAGTCAGTTTGGCTC 3’ |
|  | R5’-3’ 5’ CTTCGGCTCTGGGAACTGTCT 3’ |
| 2 | F5’-3’ 5’ AAGAGCGGCCCAAGTAGA 3’ |
|  | R5’-3’ 5’ GGACAGAACATCCCCAGG 3’ |
| 3 | F5’-3’ 5’ AACAATGGTGGGGCAGGAAT 3’ |
|  | R5’-3’ 5’ TTGGTTGAAGATCAGAGCGAGG 3’ |
